# Demagnetization Parameters Evaluation of Magnetic Shields Based on Anhysteretic Magnetization Curve

**DOI:** 10.3390/ma16155238

**Published:** 2023-07-26

**Authors:** Jianzhi Yang, Minxia Shi, Xu Zhang, Yuzheng Ma, Yijin Liu, Shuai Yuan, Bangcheng Han

**Affiliations:** 1 Institute of Large-Scale Scientific Facility and Centre for Zero Magnetic Field Science, Beihang University, Beijing 100191, China; yangjianzhi@buaa.edu.cn (J.Y.);; 2 National Institute of Extremely-Weak Magnetic Field Infrastructure, Hangzhou 310051, China

**Keywords:** magnetic shield, demagnetization, anhysteretic magnetization curve

## Abstract

To achieve the nearly zero-field environment, demagnetization is an indispensable step for magnetic shields composed of high-permeability material, which adjusts the magnetization of the material to establish magnetic equilibrium with the environmental field and improve the shielding performance. The ideal demagnetization can make the high-permeability material on the anhysteretic magnetization curve to have a higher permeability than on the initial magnetization curve. However, inappropriate parameters of degaussing field cause the magnetization state to deviate from the anhysteretic magnetization curve. Therefore, this article proposes a new assessment criterion to analyze and evaluate the parameters of degaussing field based on the difference between the final magnetization state after demagnetization and theoretical anhysteretic state of the shielding material. By this way, the magnetization states after demagnetizations with different initial amplitude, frequency, period number and envelope attenuation function are calculated based on the dynamic Jiles–Atherton (J–A) model, and their magnetization curves under these demagnetization conditions are also measured and compared, respectively. The lower frequency, appropriate amplitude, sufficient period number and logarithmic envelope attenuation function can make the magnetization state after demagnetization closer to the ideal value, which is also consistent with the static magnetic-shielding performance of a booth-type magnetically shielded room (MSR) under different demagnetization condition.

## 1. Introduction

In recent decades, the development of increasingly sensitive magnetometers [1,2] has led to numerous applications of extremely weak magnetic field measurement in fundamental physics [3,4] as well as medical and biomedical science [5,6]. Magnetic shields can provide the nearly zero-field environments with small residual field and low field gradient, which is a prerequisite for the above research [7,8]. Common configurations of magnetic shields mainly include cubic MSR [9,10] and cylindrical magnetic shield [11,12], which is composed of high-permeability soft magnetic material like permalloy and high-conductivity material like copper [13,14]. The great shielding effect can effectively reduce residual field and magnetic noise and obviously improve the signal-to-noise ratio of biological magnetic field measurement represented by magnetocardiogram (MCG) and magnetoencephalogram (MEG) [15,16]. This is mainly determined by structure and the magnetic properties of the high-permeability material. There as already some ultra-high-performance MSR used in the field of biomagnetic measurement, such as BMSR-2 [17] and the MSR built by Technical University Munich [7,8], which are composed of many shielding layers. However, due to the limitation of cost, the shielding performance cannot be improved by increasing the amount of magnetic-shielding material blindly. In this case, the magnetic properties of the material can be improved by the electromagnetic excitations, such as demagnetization [18,19] and magnetic shaking [20,21].

Demagnetization, also called degaussing, is a common technique used in many fields of science and engineering, which can be achieved by using an initially strong AC field with gradually decreasing amplitude or cooling form above the Curie temperature [22]. Its aim is always to remove the remanence within the material by destroying every magnetic order and cause a random spatial orientation of the magnetic moment. However, for magnetic shields, demagnetization cannot reduce the remanence of the soft magnetic material to zero in the geomagnetic environment, but re-establishes a new equilibrium state with the environmental field [23]. After ideal demagnetization, the shielding material is on the anhysteretic magnetization (AM) curve [24], also known as ideal magnetization curve, which has an initial permeability much higher than the initial magnetization (IM) curve. Therefore, the magnetic shields after demagnetization have better static magnetic-shielding performance, and the residual field and field gradient can be reduced significantly.

The demagnetization parameters have been studied and optimized to reduce the residual field and gradient in magnetic-shielding devices. In 2007, Thiel et al. proposed the logarithmic attenuation function to replace the common linear attenuation function, which can increase the duration and period number of the degaussing field in irreversible region and improve the demagnetization effectiveness [25]. Later, Thiel et al. evaluated the appropriate frequency of degaussing field from magnetic permeability data and suggested that a decrease in frequency can further improve the demagnetization result [26]. In 2008, Knappe-Grueneberg et al. demagnetized the BMSR-2 by arranging demagnetizing coils at the 12 edges, but the residual field still reflected the image of the recent coil, which indicates that the demagnetization procedure does not generate a random distribution of the magnetic domains and the parameters should be improved further [27]. In 2013, Voigt et al. applied a degaussing field with the larger initial amplitude, small step size between two neighboring peak values and the DC filter to reduce the residual field left inside the MSR [28]. In 2015, Altarev et al. proposed that the static residual field within MSR is dominated rather by the remanence of the shielding material and further clarified that the essence of demagnetization is to establish the magnetic equilibrium with the external condition [23]. In 2016, Sun et al. describe the magnetic equilibration procedure of demagnetization based on the dynamic J–A model and the empirical phase shift model [29]. Later, Sun et al. proposed the AM curve model to evaluate the limit of residual field inside MSR after demagnetization and achieved the excellent shielding performance with residual field below 0.13 nT inside the MSR based on the distributed demagnetization coils [24]. However, the residual field inside magnetic shields, as a comprehensive index to assess the static-shielding performance, is affected by many factors besides demagnetization, such as the gaps caused by machining errors and the structure of demagnetization coil, which cannot directly evaluate the demagnetization parameters. Therefore, a new method and assessment criterion should be adopted to evaluate the effect of different demagnetization parameters accurately.

In this article, a new method for evaluating the demagnetization effectiveness is proposed from the perspective of the magnetization state of the magnetic-shielding material after demagnetization. In this method, the difference between the magnetization state after demagnetization and the ideal anhysteretic state of the magnetic-shielding material is selected as the criterion to analyze and evaluate the influence of different demagnetization parameters. The changes of magnetization state under the degaussing fields with different frequency, initial amplitude, period number and envelope attenuation function are calculated based on the dynamic J–A model, and the demagnetization effectiveness is characterized by the difference between the end state after demagnetization and the theoretical state of AM curve (Section 2). Then, the end states of permalloy sample after demagnetizations with different parameters are tested and compared with the theoretical AM curve (Section 3). Finally, the different demagnetization parameters are applied to a booth-type MSR, and their internal state residual fields are tested (Section 4).

## 2. Evaluation Method of Demagnetization Parameters

### 2.1. Principle of Demagnetization

The traditional definition of demagnetization is the elimination of remanence inside a magnetic material. However, for the high-permeability material used for magnetic shielding, the remanence cannot be eliminated in geomagnetic environment. Actually, demagnetization can effectively improve the shielding performance of static magnetic field through re-establishing the equilibrium between the magnetization of magnetic-shielding material and the static component of environmental field [23,24]. For the magnetic-shielding devices, the common electromagnetic demagnetization method is to introduce the magnetic field with attenuated AC waveform into the magnetic-shielding layers composed of the high-permeability material through demagnetization coil. The degaussing field causes the magnetic domains of the material to oscillate repeatedly, overcome the loss of the pinning effect constantly, and finally be close to the state with the lowest potential energy, the anhysteretic state. This process can be described by the dynamic J–A model [30], which includes the hysteresis loss and eddy current loss. The differential equation form of this model can be expressed as

(1) $$\begin{array}{c} \left(\frac{\mu_{0}^{2}d^{2}}{2\rho \beta }\frac{dH}{dt}\right){\left(\frac{dM}{dH}\right)}^{2}+\left\{\delta k-\alpha \left[\left(M_{an}-M\right)+c\delta k\frac{dM_{an}}{dH_{e}}\right]\right\}\\ \cdot \frac{dM}{dH}-\left[\left(M_{an}-M\right)+c\delta k\frac{dM_{an}}{dH_{e}}\right]=0 \end{array}$$

where *M_s_* is the magnetization at saturation, *a* is the domain wall density, *k* is the pinning parameter indicating the average energy to overcome a pinning set, *α* is a mean field parameter representing the coupling of domain and *c* is the domain flexing constant. These five parameters determine the magnetic characteristics of the material. *μ*_0_ is the permeability of vacuum, *H_e_ = H + αM* represents the effective field, *δ* is the direction parameter used to ensure that the pinning opposes change in magnetization, *ρ* is the resistivity in Ω∙m, *d* is the cross-sectional dimension in meters and *β* is a geometrical factor. *M_an_* is the anhysteretic magnetization calculated by

(2) $$M_{an}=\coth\left(\frac{H_{e}}{a}\right)-\frac{a}{H_{e}}$$

**Figure 1:**
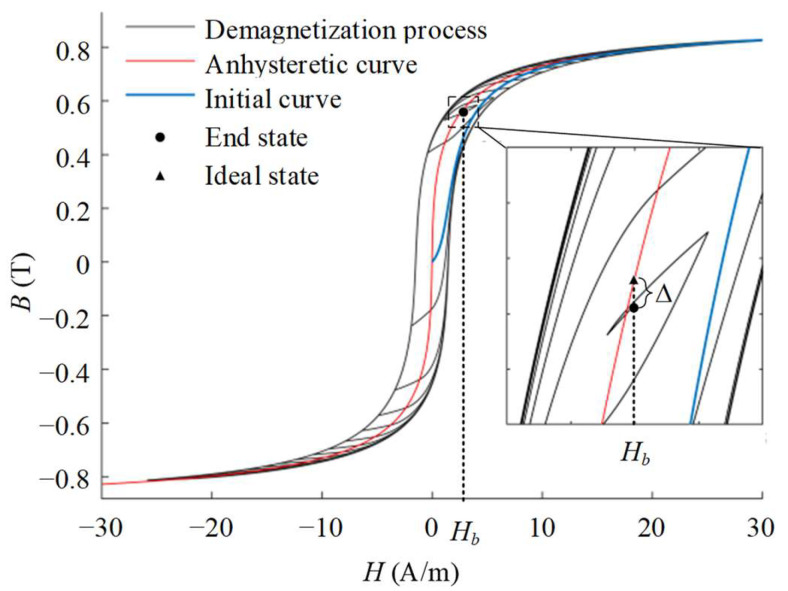
The change in magnetization state during demagnetization.

In the calculation of the anhysteretic magnetization, the effective field should be replaced by *H_e_* = *H* + *αM_an_*. The dynamic Jiles–Atherton (J–A) model is utilized to describe the demagnetization principle and characterize the change in the magnetization state during the process as shown in Figure 1. Under the degaussing field, the magnetic flux density inside the material presents a spiral attenuation, and finally lies near the AM curve corresponding to the environmental field *H_b_*. However, in the actual situation, the end state of the demagnetization is slightly lower than the ideal state of the AM curve due to the limitation of the electromagnetic demagnetization method. Their difference *∆* can be utilized as the assessment method of the demagnetization effectiveness. After demagnetization, the closer the magnetization state of the material is to the anhysteretic magnetization curve, the higher the permeability is, and the better the demagnetization effectiveness is.

### 2.2. Theoretical Calculation of Magnetization under Different Demagnetization Conditions

According to the envelope attenuation function, the common waveforms of degaussing field mainly include linear, second-order and logarithmic attenuation [19,25], which can be expressed as follows, respectively.

(3) $$H\left(t\right)=H_{0}\left(1-\frac{ft}{N}\right)\sin\left(2\pi ft\right)+H_{b}\;t\leq N/f$$

(4) $$H\left(t\right)=H_{0}{\left(1-\frac{ft}{N}\right)}^{2}\sin\left(2\pi ft\right)+H_{b}\;t\leq N/f$$

(5) $$\begin{array}{c} H\left(t\right)=H_{0}\frac{\left[a_{0}-\ln\left(a_{1}-1+ft/N\right)+\ln\left(-a_{2}+1-ft/N\right)\right]}{a_{3}}\\ \cdot \sin(2\pi ft)+H_{b}\;t\leq N/f \end{array}$$

where *H*_0_, *f* and *N* are the initial amplitude, frequency and period number of the degaussing field, respectively. In the logarithmic attenuation function, $a_{0}=\ln\left(-a_{1}/a_{2}\right)$, $a_{1}=1.001$, $a_{2}=-3$ and $a_{3}=a_{0}-\ln\left(\frac{a_{1}-1}{1-a_{2}}\right)$ are constant terms used to adjust the rate of decay. The attenuation function, initial amplitude *H*_0_, frequency *f* and period number *N* of degaussing field can be analyzed and evaluated according to the difference between the end state after demagnetization and ideal state on the AM curve.

Firstly, the demagnetization processes of permalloy with different attenuation function are calculated based on the dynamic J–A model. The five parameters of the J–A model are identified by the measured magnetization curve of permalloy as listed in Table 1 [31]. The resistivity *ρ* is set to 6.25 × 10^−6^ Ω∙m, the cross-sectional dimension *d* is 0.005 m and the geometrical factor *β* is set to 16 in this paper. In this calculation, the waveform parameters of degaussing field are set to *H*_0_ = 10 A/m, *f* = 10 Hz and *N* = 100 as reference, and the bias field *H_b_* = 0.1 A/m is used to simulate the corresponding field intensity of environmental static field. In order to simulate the accidentally magnetized state of magnetic shields, a changing field that first reaches −10 A/m and then is back to *H_b_* is added before degaussing field as the magnetization history.

**Figure 2:**
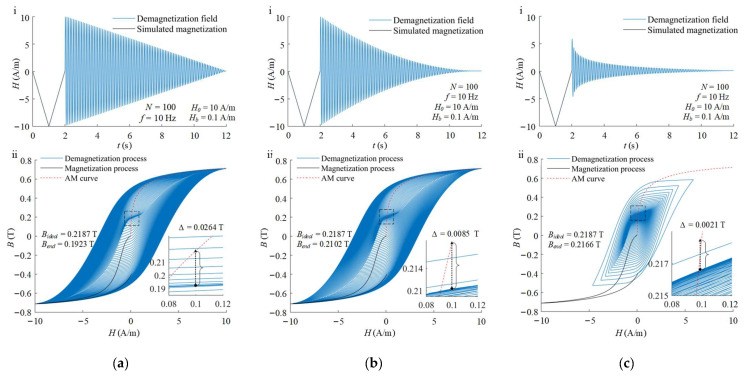
Degaussing field waveforms (**i**) and change in magnetic flux density (**ii**) during demagnetization with (**a**) linear attenuation, (**b**) second-order attenuation and (**c**) logarithmic attenuation.

The different envelope attenuation functions of degaussing field change the energy distribution in the reversible and irreversible range of the shielding material during demagnetization. The waveforms of degaussing field with different envelope attenuation function and the calculated results are illustrated in Figure 2. The simulated magnetization, demagnetization processes and AM curve are represented by the black lines, blue lines and red dotted lines, respectively. Since the logarithmic attenuation function decays quickly, the first period of degaussing field is smaller than that of the other two waveforms with the same parameter of initial amplitude *H*_0_. After demagnetizations with the linear, second-order and logarithmic attenuation function, the magnetic flux density within the material are 0.1923 T, 0.2102 T and 0.2166 T corresponding to the bias field of *H_b_*, respectively. The ideal state on the AM curve reaches 0.2187 T calculated by Equation (2). The degaussing field with logarithmic attenuation function is mainly concentrated in the small amplitude oscillation for the most of time, so that the decay step between the last periods is smaller compared to the other attenuation functions. Therefore, its end state is closer to the ideal state, meaning a better demagnetization effectiveness.

**Figure 3:**
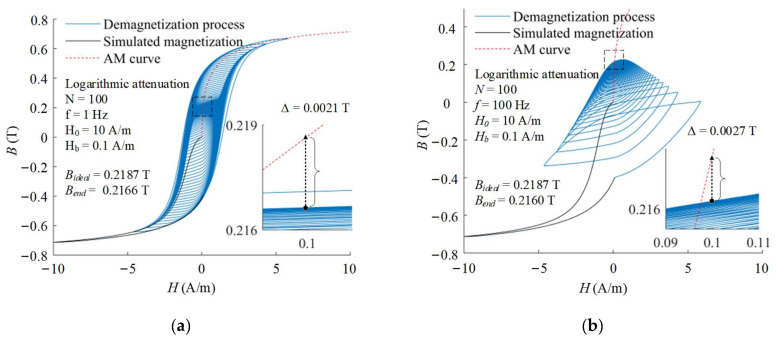
Demagnetization processes with logarithmic attenuation function and different frequency of (**a**) *f* = 1 Hz; (**b**) *f* = 100 Hz.

**Figure 4:**
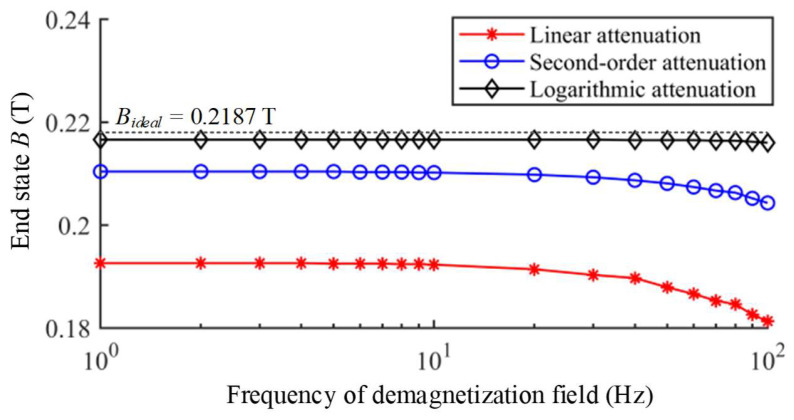
The end states of demagnetizations with different frequency *f*.

The degaussing field with higher frequency causes the increase in the eddy current loss, thus affecting the demagnetization effectiveness. The demagnetization processes with different frequency and attenuation function are also calculated by the same method. The higher frequency of degaussing field, the more eddy current losses need to be overcome during demagnetization. Taking the logarithmic attenuation function as an instance, the demagnetization processes with different frequency of *f* = 1 Hz and *f* = 100 Hz are illustrated in Figure 3. After demagnetizations of 1 Hz and 100 Hz, their end states reach 0.2166 T and 0.2160 T, respectively. With the same amplitude and period number, the excitation of magnetic flux density by 100 Hz degaussing field is less than 1 Hz, which leads to a lower efficiency in causing the magnetization state to converge into the AM curve. The end states of demagnetizations with other attenuation functions and frequencies are also calculated as shown in Figure 4. With the increase in frequency, the demagnetization effectiveness of all types of attenuation functions become worse.

**Figure 5:**
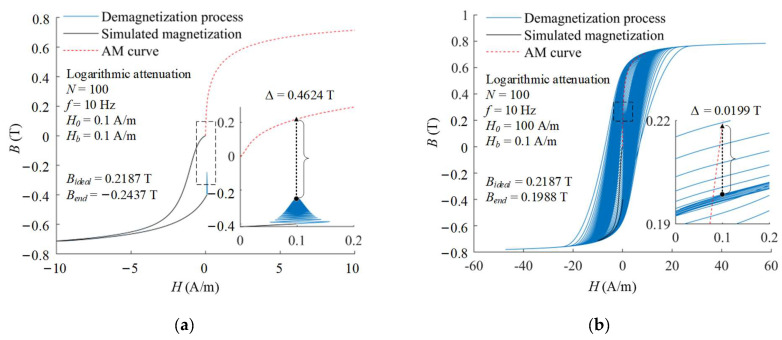
Demagnetization processes with logarithmic attenuation function and different initial amplitude of (**a**) *H*_0_ = 0.1 A/m and (**b**) *H*_0_ = 100 A/m.

**Figure 6:**
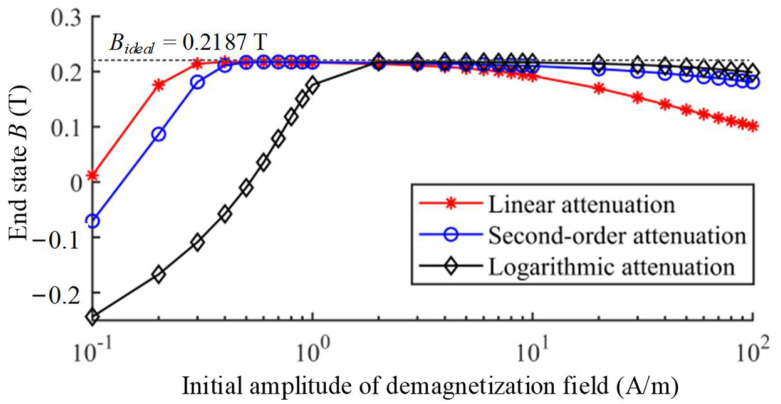
The end states of demagnetizations with different initial amplitude *H*_0_.

The initial amplitude of degaussing field is a crucial factor to overcome the unexpected magnetization of the magnetic-shielding material. The influence of the initial amplitude on the demagnetization effectiveness is also researched. The demagnetization processes with logarithmic attenuation function and different initial amplitude of *H*_0_ = 0.1 A/m and *H*_0_ = 100 A/m are illustrated in Figure 5. After demagnetizations, their end states reach −0.2437 T and 0.1988 T, respectively. Too small initial amplitude of 0.1 A/m cannot overcome the obstacle of pinning point, resulting in poor demagnetization effectiveness. The initial amplitude of 100 A/m is sufficient to overcome the pinning effect, but result in larger attenuation steps in the last periods. Therefore, a very large initial amplitude also causes the end state after demagnetization to be slightly away from the ideal state. The calculated results of other attenuation functions with different initial amplitude are shown in Figure 6. As the initial amplitude increases, the magnetic flux density of end state increases rapidly first and then decreases slowly.

**Figure 7:**
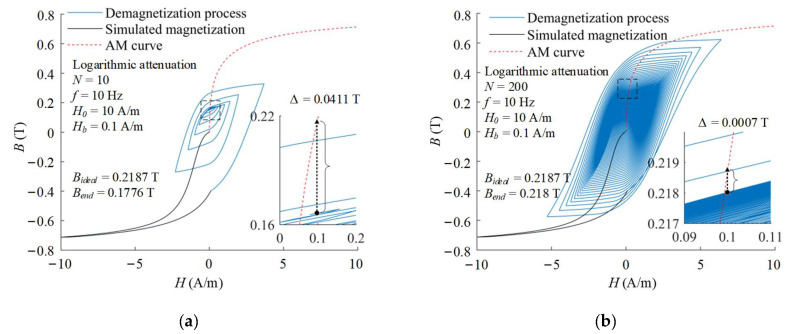
Demagnetization processes with logarithmic attenuation function and different period number of (**a**) *N* = 10 and (**b**) *N* = 200.

**Figure 8:**
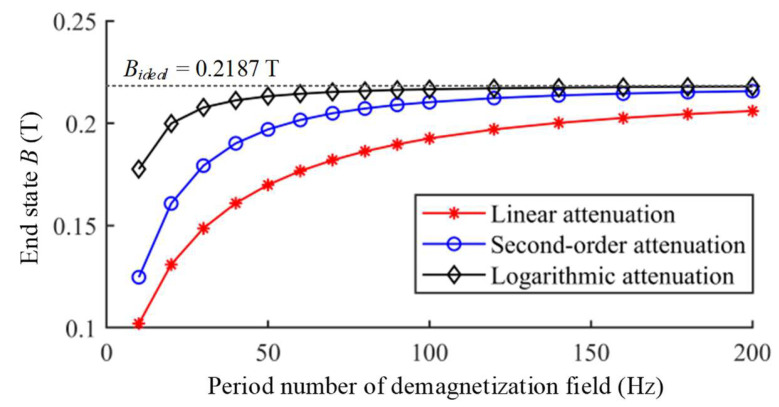
The end states of demagnetizations with different period number *N*.

More demagnetization periods can make the shielding material closer to the magnetic equilibrium and achieve better demagnetization effectiveness. It can be seen in Figure 7 that the end states after the degaussing fields with different period number of 10 and 200 reach 0.1776 T and 0.218 T, respectively. Under the conditions of suitable frequency and initial amplitude, enough demagnetization periods can make the end state basically consistent with the ideal state. For the logarithmic attenuation, when the period number is more than 100, the end magnetization state reaches 99% of the theoretical value, which can be considered as basically achieving the ideal demagnetization effectiveness. The demagnetization effectiveness of other attenuation functions with different period number are also calculated as shown in Figure 8. With the increase in the period number, the end state after demagnetization of different attenuation functions all tend to be on the AM curve corresponding to the bias field.

## 3. Measurement of Magnetization after Demagnetization with Different Parameters

**Figure 9:**
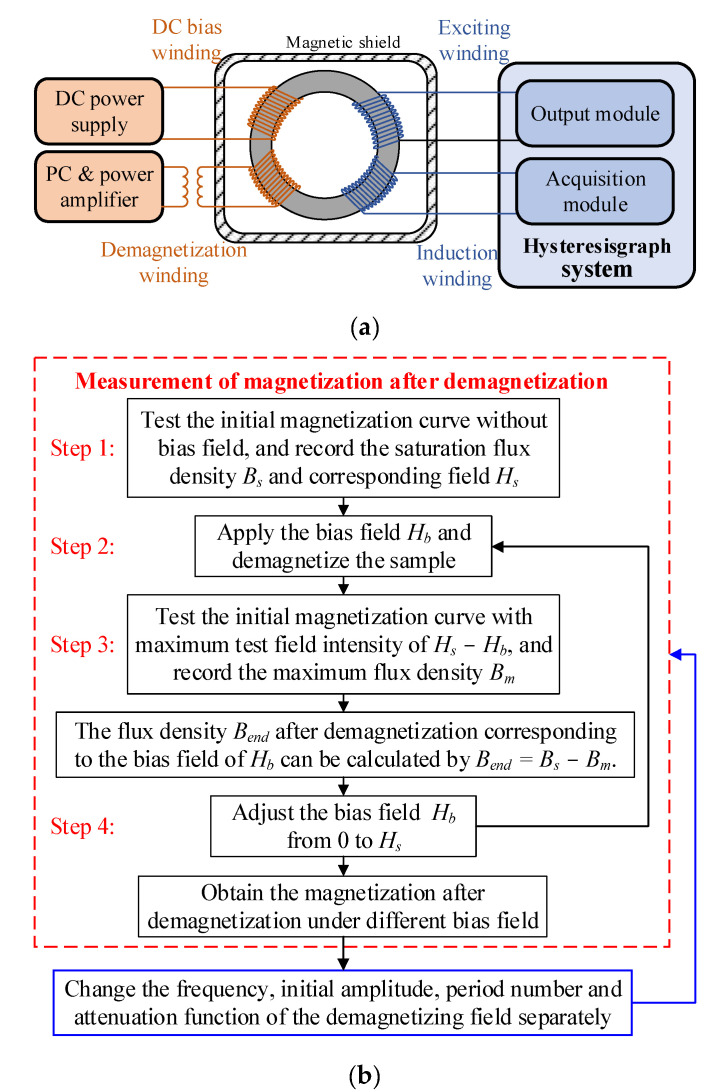
Measurement system of the magnetization state after demagnetization with different parameters: (**a**) schematic diagram and (**b**) test flowchart.

In order to test the end states after demagnetizations with different parameters, a measurement system for the magnetic properties of the ring-shape sample made of 1J85 permalloy is established as shown in Figure 9a. The outer diameter, inner diameter and thickness of the ring-shape sample are 40 mm, 32 mm and 10 mm, respectively. This system consists of the hysteresisgraph system (MATS-2010SD, LINKJOIN) to provide the excitation signal and record the induced voltage, the DC power supply (PWS4205, Tektronix, Beaverton, OR, USA) to provide the bias field and the power amplifier (7234, AE TECHRON, Elkhart, IN, USA) controlled by a PC to apply the degaussing field with different parameters. The IM curve and saturation flux density can be tested by this hysteresisgraph system. To avoid the interference of environmental field, the sample is placed in a magnetic-shielding device. Due to the drift-adjusting function of the hysteresisgraph system, the magnetic flux density of the end state after demagnetization cannot be tested directly. Therefore, using the constant saturation flux density as the benchmark, the magnetic flux density after demagnetization can be obtained by subtracting the saturation values with and without the DC bias field [32,33]. The detail test steps of the end state are as follows.

Step 1: Test the IM curve and hysteresis loop of the sample, and record its saturation flux density *B_s_* and corresponding field intensity *H_s_*_._

Step 2: Apply a DC bias field of *H_b_* to the sample through the DC power supply. Then, demagnetize the sample and test its IM curve again. In this measurement, the maximum test field intensity is set to *H_s_* − *H_b_* to ensure the same test condition as the step 1, and the maximum magnetic flux density *B_m_* is recorded.

Step 3: The flux density after demagnetization *B_end_* corresponding to the DC bias field of *H_b_* can be calculated by *B_end_* = *B_s_* − *B_m_*.

Step 4: Changing the bias field *H_b_* and repeating the step 2 and step 3, the magnetization curve of the end states after demagnetization can be measured.

Adjusting the demagnetization parameters separately, the end states are tested under different frequency, initial amplitude, period number and attenuation function of degaussing fields, which is used to evaluate the final effect of demagnetization. Under the same bias field, the higher the flux density of the material after demagnetization is, the closer the end state is to the theoretical anhysteretic magnetization curve. The whole test flow is shown in Figure 9b. Note here that the theoretical anhysteretic magnetization curve cannot be tested due to the impossibility of ideal demagnetization, which is calculated by the anhysteretic function of the fitted J–A model.

**Figure 10:**
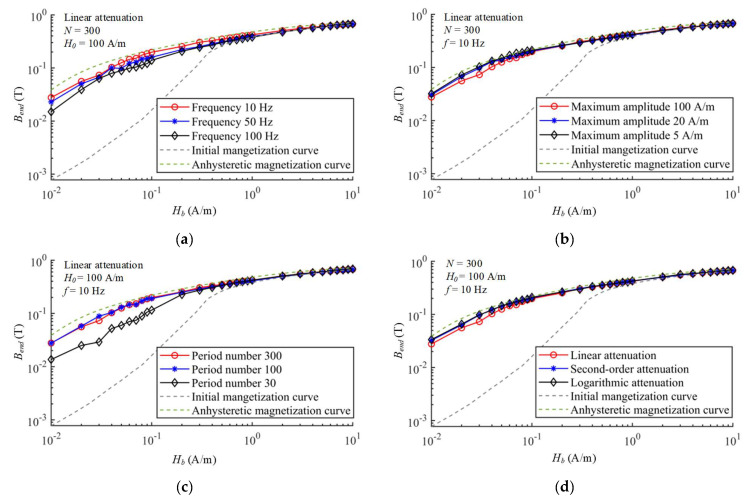
The magnetization curves of permalloy sample after demagnetizations with different (**a**) frequency, (**b**) initial amplitude, (**c**) period number and (**d**) attenuation function.

The end states after demagnetization with different parameters are shown in Figure 10. The demagnetization parameters of frequency of *f* = 10 Hz, initial amplitude of *H*_0_ = 100 A/m, period number of *N* = 300 and linear attenuation function are considered as a benchmark. The equivalent field intensity of the geomagnetic field in the shielding material is tiny and changes with the magnetization state during the demagnetization process, and its approximate range is from 0.01 A/m to 0.1 A/m according to the permeability, which can be calculated by the finite element method. Therefore, the initial stages of these curves, such as bias field *H_b_* = 0.01 A/m and *H_b_* = 0.1 A/m, are mainly selected as the comparison. The higher flux density after demagnetization indicates that the demagnetization effectiveness is better. Firstly, the magnetization curves of the end states under the degaussing fields with different frequency are illustrated in Figure 10a. The curve tested under 10 Hz degaussing field has higher magnetic flux density compared with 50 Hz and 100 Hz degaussing fields, which is closer to the theoretical AM curve. As listed in Table 2, the magnetization states under the degaussing fields with 10 Hz, 50 Hz and 100 Hz frequency can reach 0.0277 T, 0.0229 T and 0.0150 T at *H* = 0.01 A/m, as well as 0.1981 T, 0.1592 T and 0.1392 T at *H_b_* = 0.1 A/m, respectively. The theoretical AM curve is also calculated based on the J–A model, and the ideal values are 0.0388 T and 0.2187 T at *H_b_* = 0.01 A/m and *H_b_* = 0.1 A/m, respectively. All the actual tested curves are lower than the theoretical values.

The influence of initial amplitude on the magnetization curves of the end states is also illustrated in Figure 10b. At *H_b_* = 0.01 A/m and *H_b_* = 0.1 A/m, the degaussing field of 5 A/m initial amplitude can reach the magnetization state of 0.0320 T and 0.2100 T, closer to the theoretical values compared with other amplitudes. With the increase in the initial amplitude, the magnetization state after demagnetization is slightly reduced. However, for magnetic-shielding device, especially the MSR with large size, the distribution of the degaussing field in the layers is often uneven, so it is necessary to use a large initial amplitude to ensure that all materials can be demagnetized.

It can be seen in Figure 10c that too few demagnetizing periods can seriously affect the magnetization state after demagnetization. Under the degaussing field with 30 periods, the magnetization states at *H_b_* = 0.01 A/m and *H_b_* = 0.1 A/m can only reach 0.0136 T and 0.1151 T, which is significantly lower than the degaussing field with 300 periods. Adjusting the period number to *N* = 100, the tested curve is basically consistent with the period number of *N* = 300. It demonstrates that when the demagnetizing period is enough, the increase in the period number has little effect on the demagnetization effectiveness.

The magnetization curves are also tested under the degaussing fields with linear, second-order and logarithmic attenuation functions as shown in Figure 10d. The tested curve under the degaussing field with logarithmic attenuation function is closer to the theoretical value of AM curve than the linear and second-order attenuation. At *H_b_* = 0.01 A/m and *H_b_* = 0.1 A/m, the magnetization states after the demagnetization of logarithmic attenuation can reach 0.0336 T and 0.2098 T, higher than 0.0324 T and 0.2051 T of the second-order attenuation as well as 0.0276 T and 0.1981 T of the linear attenuation.

## 4. Demagnetization Experiment of MSR

**Figure 11:**
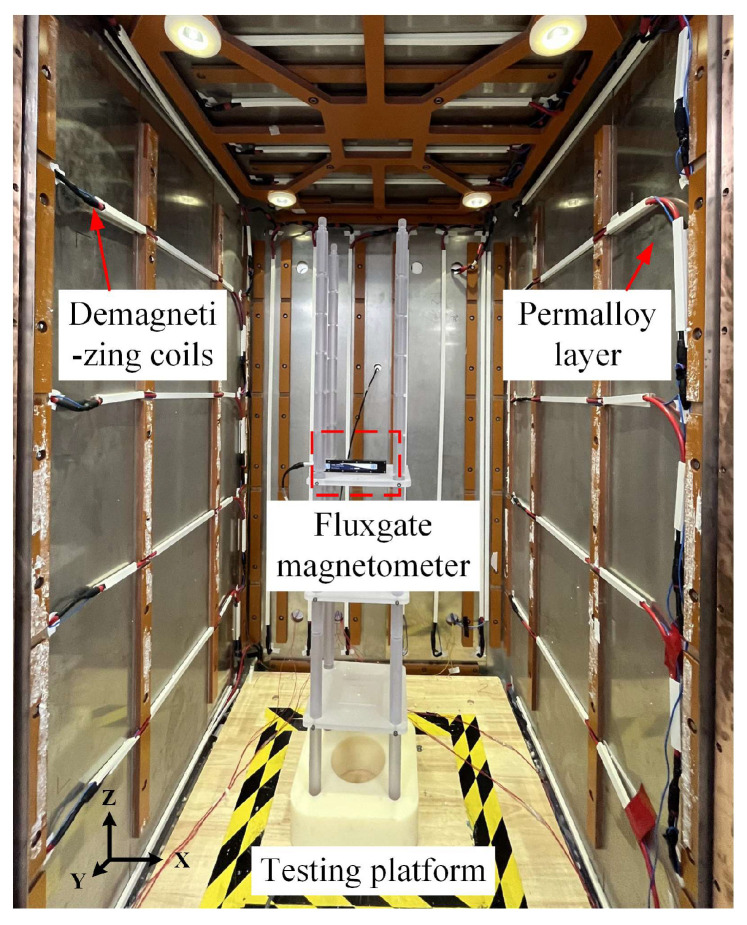
Measurement of residual field inside the booth-type MSR.

The static residual fields inside a booth-type MSR are tested to characterize its static magnetic-shielding performance after demagnetizations with different parameters. This MSR is composed of two permalloy layers with 3 mm thickness and one aluminum layer with 5 mm thickness. Its internal space size is 1050 mm × 1050 mm × 1900 mm. The fluxgate magnetometer (MAG-13, Bartington Instruments, Witney, UK) with the test range of 60 μT and noise floor of 10 pT/Hz^1/2^ is placed on a nonmagnetic test platform to measure the residual field inside the MSR as shown in Figure 11. The zero offset of the fluxgate magnetometer is corrected by testing the two opposite directions at the same location. The central area of 600 mm × 600 mm × 600 mm is divided into 3 × 3 × 3 grids to characterize the distribution of residual field. The test signals are converted to digital signals and collected by the data acquisition module (NI-USB6366). To ensure the same magnetization state of the shielding material, the MSR are artificially magnetized by introducing a direct current of 1 A into the demagnetizing coils before demagnetization.

**Figure 12:**
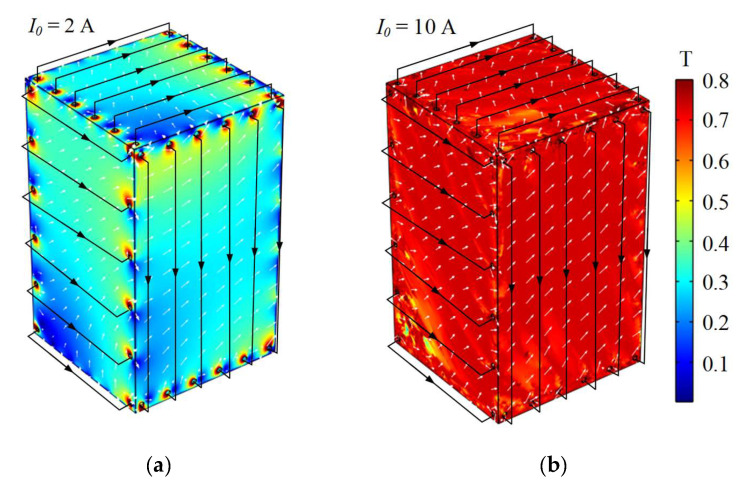
Simulation of the distributions of degaussing fields inside shielding layer of (**a**) 2 A and (**b**) 10 A excited currents.

The demagnetization coils are wound on each permalloy layer using distributed winding, which can generate uniform degaussing field within the shielding layers compared with the common I-coil and L-coil [24]. Based on COMSOL Multiphysics 6.0 software, the distribution of the degaussing field inside the outermost shielding layer is calculated based on Maxwell’s equations. In simulation, the geometric model of the outermost permalloy layer is established according to the actual size of the MSR, and the measured IM curve is select as the magnetic property of the shielding material. Due to the very thin thickness of the shielding layer, this domain is divided into free tetrahedral meshes with the minimum size of 0.5 mm. The wires of the demagnetizing coils as calculated as “edge current” module, and the excited current values are set to 2 A and 10 A, respectively. In order to reduce the influence of the magnetic insulating boundary condition, an infinite element domain is established on the outermost surface of the computational domain. It can be seen in Figure 12 that, under the current of 2 A, the degaussing field is unevenly distributed in the shielding layer, especially at the edges and corners. Setting the current to 10 A, most areas of the shielding layer reach the magnetic saturation.

**Figure 13:**
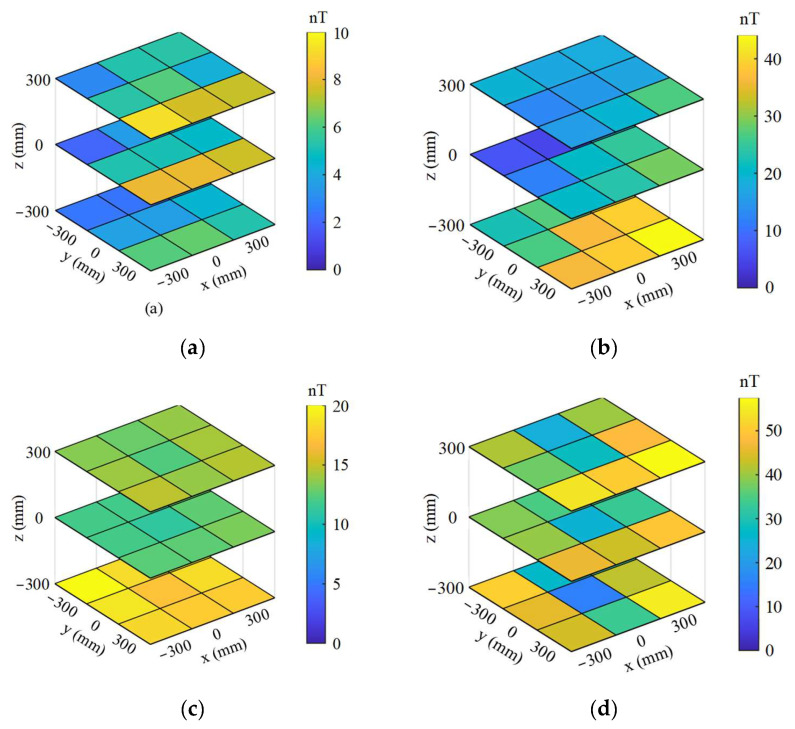
Distribution of residual field inside the booth-type MSR after demagnetization with logarithmic attenuation envelope function and different other parameters: (**a**) *f* = 10 Hz, *N* = 100, *I*_0_ = 10 A; (**b**) *f* = 10 Hz, *N* = 100, *I*_0_ = 2 A; (**c**) *f* = 50 Hz, *N* = 100, *I*_0_ = 10 A; (**d**) *f* = 10 Hz, *N* = 10, *I*_0_ = 10 A.

The distributions of static residual fields at the central position of the MSR after demagnetization with different initial amplitude *H*_0_, frequency *f*, period number *N* and attenuation function are tested as listed in Table 3. In this measurement, the demagnetization parameter of initial amplitude is replaced by the initial amplitude of the demagnetization current *I*_0_. With the demagnetization parameters of *f* = 10 Hz, *N* = 100 and *I*_0_ = 10 A, the central residual fields corresponding to the linear, second-order and logarithmic attenuation envelope functions are 5.5 nT, 5.2 nT and 5.2 nT, respectively. The different attenuation envelope functions have little effect on the residual field inside the MSR, which also indicates that it is not sufficient to judge the demagnetization effectiveness only from the perspective of the residual field. Taking this set of demagnetization parameters as a reference, the residual fields in the MSR increase obviously when the initial amplitude of the demagnetization current is set to 5 A. Due to the uneven distribution of the degaussing field, the amplitude of degaussing field inside parts of the layers is too small to overcome the accidental magnetization. Increasing the frequency of degaussing field and decreasing the number of demagnetizing periods both lead to the degradation of static-shielding performance. The change trends of residual fields in the MSR under different demagnetization parameters are basically consistent with the theoretical calculation and the actual measurement of the magnetization state after demagnetization. The distributions of the residual field after demagnetization with different parameters and logarithmic attenuation envelope function are tested as shown in Figure 13. It can be seen that, in addition to demagnetization, the gaps between the door and the shielding layer along y direction also cause a significant impact on the residual field.

## 5. Conclusions

From the perspective of AM curve, a new assessment criterion is proposed to analyze and evaluate the parameters of degaussing field based on the difference between the magnetization state after demagnetization and theoretical anhysteretic state, including initial amplitude, frequency, period number and envelope attenuation function. Through the theoretical calculation based on dynamic J–A model, it can be found that the low frequency, appropriate amplitude, sufficient period number and logarithmic envelope attenuation function of degaussing field lead to the final magnetization state close to the corresponding anhysteretic state, which is basically consistent with the measurement of the end states under different demagnetization condition. Although the calculation results show that a very large initial amplitude causes a decrease in the final magnetization state, considering the uneven distribution of degaussing field inside shielding layers, the demagnetization parameters with sufficient initial amplitude and more period number should be used in practical application. The appropriate parameters are applied to the demagnetization of the booth-type MSR to reduce its internal residual field from 25.1 nT to 5.2 nT. This evaluation method provides a new possibility to further optimize the demagnetization parameters from the perspective of the magnetization state of the shielding material.

## Figures and Tables

**Table 1:** The Jiles–Atherton model parameters of permalloy.

Parameter	***M_s_***	***c***	***k***	***a***	***α***
value	$6.400\times 10^{5}$ A/m	1.577 A/m	0.207	1.253 A/m	$7.175\times 10^{-5}$

**Table 2:** Magnetization state of permalloy sample under different demagnetization parameters.

Demagnetization Parameters	Magnetic Flux Density (@0.01 A/m)	Magnetic Flux Density (@0.1 A/m)
10 Hz frequency	0.0277 T	0.1981 T
50 Hz frequency	0.0229 T	0.1592 T
100 Hz frequency	0.0150 T	0.1392 T
100 A/m initial amplitude	0.0277 T	0.1981 T
20 A/m initial amplitude	0.0305 T	0.2034 T
5 A/m initial amplitude	0.0320 T	0.2100 T
300 period number	0.0277 T	0.1981 T
100 period number	0.0276 T	0.1899 T
30 period number	0.0136 T	0.1151 T
Linear attenuation	0.0277 T	0.1981 T
Second-order attenuation	0.0324 T	0.2051 T
Logarithmic attenuation	0.0336 T	0.2098 T
AM curve (theoretical value)	0.0388 T	0.2187 T

**Table 3:** The static residual fields at the central position of the MSR under different demagnetization parameters.

Parameters	Linear Attenuation	Second-Order Attenuation	Logarithmic Attenuation
*f* = 10 Hz, *N* = 100, *I*_0_ = 10 A	5.5 nT	5.2 nT	5.1 nT
*f* = 10 Hz, *N* = 100, *I*_0_ = 2 A	20.4 nT	19.5 nT	20.8 nT
*f* = 50 Hz, *N* = 100, *I*_0_ = 10 A	16.8 nT	16.1 nT	11.2 nT
*f* = 10 Hz, *N* = 10, *I*_0_ = 10 A	21.4 nT	19.4 nT	25.1 nT

## Data Availability

The data presented in this study are available on request from the corresponding author.
